# Volatile Components of *Haplophyllum canaliculatum* Boiss. by Different Extraction Procedures

**DOI:** 10.1155/2020/4202871

**Published:** 2020-05-30

**Authors:** Nastaran Ahadi, Marzieh Torabbeigi, Zahra Aghaiee Meibodei, Fatemeh Safatian

**Affiliations:** ^1^Pharmaceutical Science Branch, Islamic Azad University, Tehran, Iran; ^2^School of Public Health and Safety, Shahid Beheshti University of Medical Sciences, P.O. Box 16858-116, Tehran, Iran; ^3^Department of Chemistry, East Branch of Tehran (Ghiam Dasht), Islamic Azad University, Tehran, Iran; ^4^Ramsar International Branch, Mazandaran University of Medical Sciences, Ramsar, Iran

## Abstract

Volatile components of *Haplophyllum canaliculatum* Boiss. grown in Iran were extracted by hydrodistillation (HD), solvent-free microwave extraction (SFME), and headspace solid-phase microextraction (HS-SPME). The components were analyzed by means of GC and GC-MS. The extraction time and temperature for HS-SPME, microwave, power, and exposure time of extraction for SFME were optimized. Twenty-five compounds that represent 99.88% of total compounds in the oil were obtained by the HD method, and the major components for this method were identified as *β*-pinene (18.90%), 1,8-cineole (13.94%), and piperitone (12.22%). However, piperitone (34.50%), caryophyllene oxide (9.94%), and *a*-eudesmol were the main compounds among twenty-one constituents, representing 99.89% of the total composition that were characterized in volatiles extracted by the SFME method. Moreover, thirteen compounds, representing 99.95% of the total constituents, were characterized in volatile fraction extracted by the HS-SPME method, which were dominated by *β*-pinene (21.13%), *a*-pinene (13.07%), limonene (11.65%), and *δ*-2-carene (10.23%) as major constituents.

## 1. Introduction

The genus *Haplophyllum* belongs to the Rutaceae family, and its eighteen species are present in Iran. These species are scattered in different regions of Iran (deserts and mountain ranges)*. H. canaliculatum*, *H. tuberculatum*, *H. perforatum*, and *H. robustum* are endemic species of Iran. Some species of *Haplophyllum* are grown in Afghanistan, Pakistan, Middle Asia, North Africa, Arabic countries, Anatolia, Iraq, and Palestine [1]. Literature survey revealed that the chemical composition of some species of *Haplophyllum* was studied previously. *β*-ocimene (12.3%) and *β*-caryophyllene (11.6%) were the main constituents in the essential oil of *H. tuberculatum* from Oman [2]. The volatile fraction of *H. tuberculatum* from the United Arab Emirate was dominated by linalool (15.0%) and linalyl acetate (10.6%) as main compounds [3]. The major constituents in the essential oil of *H. linifolium* from Spain which was characterized were *β*-caryophyllene (20.64%), bicyclogermacrene (14.73%), and *d*-cadinene (13.40%) [4]. Palmito-*γ*-lactone (45.8%) and octadecatrienoic acid (10.7%) were the main compounds in the essential oil of *H. megalanthum* reported from Turkey [5]. GC-MS analysis of *H. canaliculatum* collected from Shiraz, Fars, Iran, showed that shoot cultures mainly contained piperitone (10.92%) and *β*-caryophyllene (12.67%) [6]. The essential oil of *Haplophyllum buhsei* Boiss. from Iran was analyzed for the first time using gas chromatography (GC) and gas chromatography–mass spectrometry (GC-MS). The major compounds were *β*-caryophyllene (12.9%), limonene (9.7%), *β*-pinene (7.9%), linalool (7.4%), *α*-pinene (6.4%), and 1,8-cineole (5.5%) [7]. *H. robustum* Bge. was isolated by hydrodistillation and was analyzed by GC-MS. Twenty-three compounds representing 86.1% of the total components were detected. The oil consisted mainly of monoterpene hydrocarbons and a small percentage of sesquiterpenes. The major compounds were sabinene (30.5%), β-pinene (18.2%), and limonene (12.1%) [8].

Although hydrodistillation has been the most prevalent method to extract the volatile fractions from the medicinal herbs or plants [9], other extraction methods for isolation of volatile fractions were developed, such as microextractions and microwave-assisted extractions [10–12].

Pawliszyn and his coworkers have introduced solid-phase microextraction (SPME), which was developed for the analysis of volatiles in plant tissues [13–19]. Some advantages of the SPME method for the analysis of volatile components are that it is time saving, solvent-free, and cost-effective and requires a small amount of samples [20–24].

Solvent-free microwave extraction is a new technique which combines microwave heating with dry distillation at atmospheric pressure for the isolation and concentration of the essential oils in plant materials. In the solvent-free microwave extraction method, there is no need to add any solvent or water if fresh plant material is used. For dry plant material, the sample is rehydrated by soaking in water for some time, followed by draining off the excess water [25].

In this paper, we report the comparative study on the volatile chemical composition of *H. canaliculatum* that are grown wild in Iran by using three methods, that is, hydrodistillation (HD), solvent-free microwave extraction (SFME), and headspace solid-phase microextraction(HS-SPME), for the first time.

## 2. Experimental

### 2.1. Plant Material

The leaves of *Haplophyllum canaliculatum* Boiss. were harvested from altitude 1300 meters of Tang Zagh mountains with 27°933′44″ latitude and 55°916′66″ longitude in Hormozgan province, Iran, in November 2013. Voucher specimens (1513-AUPF) have been deposited at the herbarium of Pharmaceutical Science Branch, Islamic Azad University Tehran, Iran. The collected tissue was dried under shade at room temperature.

### 2.2. Volatile Compounds Extraction

#### 2.2.1. Hydrodistillation

50 g of air-dried *H. canaliculatum* leaves were grounded and subjected to hydrodistillation using a Clevenger-type apparatus for 4 h. The obtained light yellowish oil was dried over anhydrous Na_2_SO_4_ and kept in 4°C for further analysis.

#### 2.2.2. Solvent-Free Microwave Extraction

The SFME method was performed using a Milestone Microsynth (Italy) microwave oven operating at 2450 MHz. The maximum power of the oven was 1000 W. 100 g fresh leaves of *H. canaliculatum* were moisturized with deionized water for 1 h. The humid plant material was heated in a microwave oven at 800 W for 25 minutes under atmospheric pressure using a Clevenger apparatus. The isolated yellowish oil was dried on anhydrous Na_2_SO_4_ and kept to be analyzed.

#### 2.2.3. Headspace Solid-Phase Microextraction

HS-SPME is an efficient method to isolate volatile organic compounds (VOCs). 2 g of powdered dried leaves of *H. canaliculatum* were placed in a 20 mL vial that was capped with the PTFE septum and placed in room temperature for 24 h (equilibrium time). A manual SPME holder with a 65 *μ*m polydimethylsiloxane (PDMS) (Supelco, USA, 2011) fiber was used for adsorbing the VOCs of *H. canaliculatum*, which conditioned in the GC injector at 250°C for 5 h. The fiber was placed in headspace of the vial for 10 minutes (extraction time). Desorption of VOCs was performed at 250°C for 2 minutes in splitless GC and GC-MS injectors. The adsorbed analytes were desorbed at 250°C for 4 minutes in splitless GC and GC-MS injectors.

### 2.3. Chemical Composition Analysis

The chemical composition of samples was analyzed by GC and GC-MS. The GC analysis was performed using Shimadzu GC-15A with a 30 m × 0.25 mm, film thickness 0.32 *μ*m DB-5 column. The initial temperature was 60°C (3 minutes hold time), then heated to 230°C as the final temperature with 5°C min^−1^ heat gradient, and kept 10 minutes in 230°C. Injector and FID detector temperatures were set at 250°C. N_2_ was used as a carrier gas (1 mL min^−1^). Quantitative data were acquired from the area percentage of each GC peak without the use of correction parameters.

GC-MS analysis was performed on a Hewlett-Packard 6890 gas chromatograph coupled with a 5973 mass selective detector, which was equipped with a HP-5MS column (30 m × 0.25 mm, film thickness 0.32 *μ*m). The program temperature was similar to the GC condition. Helium (99.999% purity) was used as a carrier gas with 1 mL min^−1^ flow rate. Mass spectra were achieved at 70 eV as ionization energy in the electron impact mode.

### 2.4. Identification of Components

Identification of constituents was performed by comparison of their mass spectra and Kovats retention indices (KI) of samples constituents, which were calculated using the homologue series on normal alkanes (C8–C20) using the Kovats equation, with those given in authentic references [26].

## 3. Result and Discussion

### 3.1. HS-SPME Optimization Procedure

#### 3.1.1. Temperature

**Figure 1:**
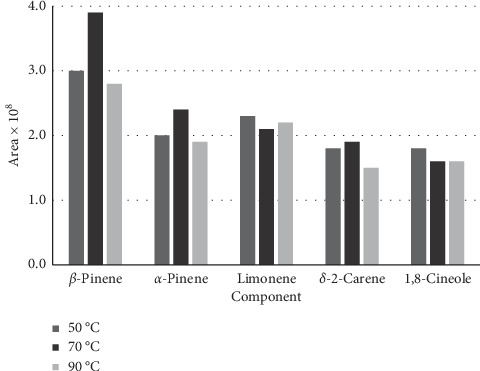
The effect of the extraction temperature on the extracted main components of *H. canaliculatum* in the HS-SPME procedure.

As HS-SPME efficiency is affected by temperature, the effect of temperature on isolation of main components, obtained by this method, was investigated. At less than 50°C, the adsorption of volatiles was not considerable, and also, on the other hand, at temperatures above 90°C, desorption of the compounds from fiber was significant. Then, the temperature range was selected as 50 to 90°C. As shown in Figure 1, the optimum temperature for leaves of *Haplophyllum canaliculatum* was 70°C.

#### 3.1.2. Exposure Time

**Figure 2:**
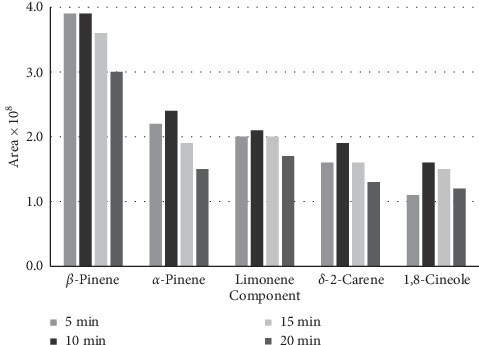
The effect of the exposure time of SPME fiber on peak areas of the main volatile compounds of *H.canaliculatum* in the HS-SPME procedure.

The optimization for exposure time was carried out for 5, 10, 15, and 20 minutes. It has been specified that 10 minutes was the optimum exposure time as shown in Figure 2. After 10 minutes of exposure time, the extraction of the main volatiles increased, and then, by increasing the exposure time, isolation of these components decreased. Desorption of volatiles from the fiber by increasing the contact time resulted in this reduction.

#### 3.1.3. Sample Weight

**Figure 3:**
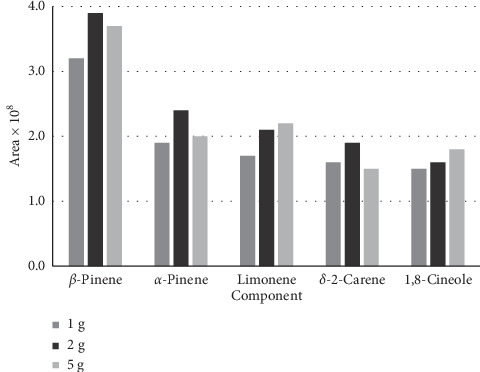
The effect of the sample weight of the plant material on main components of *H. canaliculatum* extracted by the HS-SPME procedure.

HS-SPME was carried out with samples of 1, 2, and 5 g, which were placed in the SPME. As shown in Figure 3, the optimum weight of leaves of *Haplophyllum canaliculatum* was 2 g. The differences between 2 and 5 g samples were negligible; therefore, 2 g samples were used in HS-SPME experiments.

It has been specified that as the temperature of the extraction increased, the rate of extraction changed as well. However, the distribution decreased constantly. Therefore, to obtain desirable sensitivity and extraction rate, an adequate temperature is needed. The amount of the volatile fractions was at its highest at the temperature of 70°C and after 10 minutes of exposure time (Figures 1 and 2). It seems that at this time, the HS-SPME process reaches equilibrium, and SPME has a maximum sensitivity at this point. Increasing the sample weights caused an increase in the concentrations of the volatile fractions in the headspace. Samples of 1, 2, and 5 g were placed in the SPME vial, and the HS-SPME process was carried out for 10 minutes at 70°C. The peak areas of the extracted main components were plotted for different weights of samples (Figure 3).

### 3.2. Optimization of SFME Conditions

#### 3.2.1. Power of Microwave

**Figure 4:**
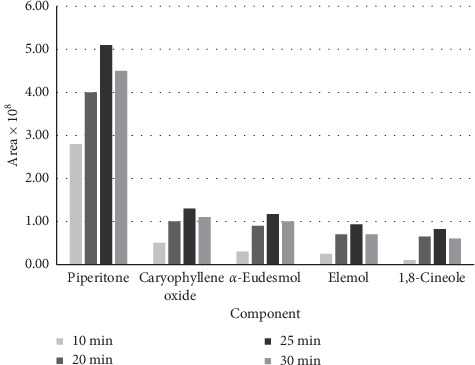
The effect of the exposure time of SFME on the isolated main volatile compounds of *H. canaliculatum*.

The optimization of microwave power was performed in 400, 600, 800, and 1000 W. As shown in Figure 4, the power of 800 W was selected as the optimum condition.

#### 3.2.2. Exposure Time

As shown in Figure 4, the optimum time for extraction of the compounds in the SFME method was 25 minutes.

**Figure 5:**
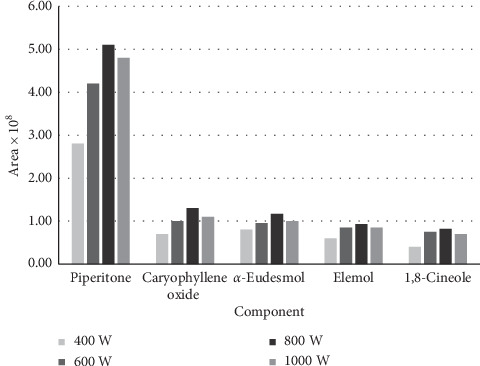
The effect of microwave power on the peak areas of the main volatile compounds of *H. canaliculatum* isolated by the SFME procedure.

To optimize the conditions, 5 main components in the essential oil were chosen. Extraction was carried out for 10, 20, 25, and 30 minutes in 400, 600, 800, and 1000 W power of microwave. The optimum time for extraction was 25 minutes (Figure 4). As shown in Figure 5, 800 W was the best microwave power of extraction for the main components.

### 3.3. Comparison of the Chemical Compositions of Essential Oils Obtained by HD, HS-SPME, and SFME

**Figure 6:**
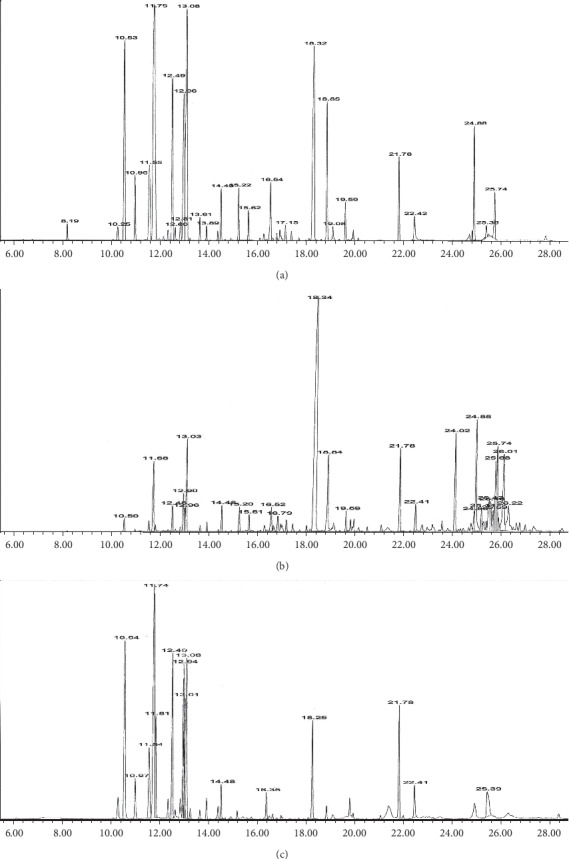
The gas chromatograms of *H. canaliculatum* extracted by (a) HS-SPME, (b) SFME, and (c) hydrodistillation methods.

The volatile composition of *H. canaliculatum* extracted by hydrodistillation (HD), solvent-free microwave extraction (SFME), and headspace solid-phase microextraction (HS-SPME) and their percentage are listed in Table 1.  Figure 6 shows the resultant gas chromatograms by these methods. As shown in Table 1, twenty-five compounds represent 99.88% of the total compounds in the oil that are obtained by the HD method, with *β*-pinene (18.90%), 1,8-cineole (13.94%), piperitone (12.22%), *α*-pinene (8.95%), and limonene (8.40%) characterized as dominate compounds. Piperitone (34.50%), caryophyllene oxide (9.94%), *α*-eudesmol (7.94%), elemol (6.32%), and 1,8-cineole (5.56%) were the main compounds among twenty-one identified constituents, which represent 99.89% of the total composition in essential oil extracted by the SFME producer. Thirteen compounds, representing 99.95% of the total constituents, were characterized in a volatile fraction extracted by the HS-SPME method, with *β*-pinene (21.13%), *α*-pinene (13.07%), limonene (11.65%), and *δ*-2-carene (10.23%) as major constituents.

## 4. Conclusion

In this investigation, the volatile constituents of *Haplophyllum canaliculatum* were extracted by hydrodistillation (HD), solvent-free microwave extraction (SFME), and headspace solid-phase microextraction (HS-SPME) methods. The essential oil obtained by the SFME method has higher percentage of oxygenated compounds, which are more odoriferous and valuable than the essential oil extracted by the HD process. SFME is a fast, eco-friendly, and low-cost method in comparison with the other extraction procedures. The usage of PDMS fiber as a nonpolar solid phase caused an increase in the extraction of nonpolar compounds compared with HD and SFME methods.

## Figures and Tables

**Table 1:** Composition of volatile fractions of *Haplophyllum canaliculatum* obtained by hydrodistillation (HD), headspace solid-phase microextraction (HS-SPME) (PDMS fiber, 70°C, time exposure = 10 minutes), and solid-phase microwave extraction (800 W time exposure = 25 minutes).

Component name		KI^*∗*^	HD	SFME		HS-SPME	
			%	%	Calculated KI	%	Calculated KI
2-Hexenal		855	0.45	ND^*∗∗*^	—	ND	—
Thujene		930	0.53	ND	—	ND	—
*α*-Pinene		939	8.95	0.67	944.78	13.07	946.33
Camphene		954	2.18	ND	—	2.40	962.93
Sabinene		975	4.11	ND	—	5.34	984.94
*β*-Pinene		979	18.9	4.11	990.34	21.13	992.66
Myrcene		991	ND	ND	—	5.32	995.36
*δ*-2-Carene		1002	6.37	1.36	—	10.23	1023.14
*α*-Terpinene		1017	0.38	ND	—	ND	—
Para-cymene		1025	0.77	ND	—	ND	—
Limonene		1029	8.4	2.25	1040.08	11.65	1041.73
*β*-Phellandrene		1030	ND	1.16	1042.56	6.58	1044.62
1,8-Cineole		1031	13.94	5.56	1045.45	8.58	1046.69
Gamma-terpinene		1060	0.61	ND	—	ND	—
Sabinene hydrate		1070	0.38	ND	—	ND	—
Linalool		1097	1.64	1.43	1105.28	1.75	1105.72
Menth-2-en-1-ol<cis-para>		1122	1.58	1.38	1137.44	ND	—
Menth-2-en-1-ol<trans-para>		1141	0.83	0.90	1155.55	ND	—
Borneol		1169	ND	ND	—	1.44	1188.10
Terpinen-4-ol		1177	2.17	1.62	1196.03	ND	—
*α*-Terpineol		1189	ND	0.99	1208.01	ND	—
Cis-piperitol		1196	0.46	ND	—	ND	—
Piperitone		1253	12.22	34.50	1281.13	5.88	1276.88
Bornyl acetate		1289	4.90	5.29	1305	ND	—
Pinocarvyl acetate		1312	0.52	ND	—	ND	—
Myrtenyl acetate		1327	1.11	1.08	1342.5	ND	—
Caryophyllene (trans)		1419	2.71	5.20	1455.02	6.58	1455.02
Elemol		1550	ND	6.32	1579.42	ND	—
Caryophyllene oxide		1583	4.14	9.94	1625.29	ND	—
Eudesmol (5-epi-7-epi-alpha)		1608	ND	1.51	1642.94	ND	—
Gamma eudesmol	1632	ND	1.42	1671.17	ND	—
Caryophylla-4 (14),8 (15)-dien5-alpha-ol		1641	1.63	5.26	—	ND	—
*α*-Eudesmol		1654	ND	7.94	1695.88	ND	—
Monoterpenes (%)			51.2	9.55		75.72	
Sesquiterpenes (%)			2.71	5.2		6.58	
Oxygenated compounds (%)			45.97	85.14		17.65	
Total identified compounds (%)			99.88	99.89		99.95	
Yield (%)			1.24	1.04		—	

^*∗*^KI: Kovats index [26]; ^*∗∗*^ND: not detected.

## Data Availability

The data used to support the findings of this study are included within the article.
